# Molecular and morphological diversity of *Zygnema* and *Zygnemopsis* (Zygnematophyceae, Streptophyta) from Svalbard (High Arctic)

**DOI:** 10.1080/09670262.2018.1476920

**Published:** 2018-10-08

**Authors:** Martina Pichrtová, Andreas Holzinger, Jana Kulichová, David Ryšánek, Tereza Šoljaková, Kateřina Trumhová, Yvonne Nemcova

**Affiliations:** a Charles University, Faculty of Science, Department of Botany, Benátská 2, 128 00Prague, Czech Republic; b University of Innsbruck, Institute of Botany, Functional Plant Biology, Sternwartestraße 15, 6020Innsbruck, Austria

**Keywords:** Arctic, chloroplast shape, cryptic diversity, microscopy, molecular phylogeny, *rbc*L, Svalbard, *Zygnema*, *Zygnemopsis*

## Abstract

Filamentous conjugating green microalgae (Zygnematophyceae, Streptophyta) belong to the most common primary producers in polar hydro-terrestrial environments such as meltwater streamlets and shallow pools. The mats formed by these organisms are mostly composed of sterile filaments with *Zygnema* morphology, but the extent of their diversity remains unknown. Traditional taxonomy of this group is based on reproductive morphology, but sexual reproduction (conjugation and formation of resistant zygospores) is very rare in extreme conditions. In the present study we gave the first record of zygospore formation in Svalbard field samples, and identified conjugating filaments as *Zygnemopsis lamellata* and *Zygnema* cf. *calosporum*. We applied molecular phylogeny to study genetic diversity of sterile *Zygnema* filaments from Svalbard in the High Arctic. Based on analysis of 143 *rbc*L sequences, we revealed a surprisingly high molecular diversity: 12 Arctic *Zygnema* genotypes and one *Zygnemopsis* genotype were found. In addition, we characterized individual Arctic genotypes based on cell width and chloroplast morphology using light and confocal laser scanning microscopy. Our findings highlight the importance of a molecular approach when working with sterile filamentous Zygnematophyceae, as hidden diversity might be very beneficial for adaptation to harsh environmental conditions, and experimental results could be misinterpreted when hidden diversity is neglected.

## Introduction

Conjugating green algae (class Zygnematophyceae) are the most species-rich group of charophyte algae, with more than 4000 described species (Gerrath, 2003). They are morphologically diverse and visually attractive. Unravelling the diversity and evolution of this group is an important research topic in current phycology, because conjugating green algae are considered to be the closest algal relatives of land plants (Wickett *et al*., 2014; Zhong *et al*., 2015), and the genetic identity of the vast majority of morphospecies is still unknown (however see e.g. Drummond *et al*., 2005; Gontcharov & Melkonian, 2005; Stancheva *et al*., 2014). Zygnematophyceae are also important ecological dominants in certain habitat types. For example, unicellular desmids typically occur in benthic communities of peat bogs (Coesel & Meesters, 2007) or ephemeral freshwater pools (Šťastný, 2008). Their species composition reflects abiotic conditions; hence they are used as bioindicators (Coesel, 2001) and model organisms in ecological studies (Svoboda *et al*., 2014). Filamentous Zygnematophyceae often dominate various freshwater habitats where they can quickly produce large amounts of biomass. They are particularly important in polar hydro-terrestrial habitats where they form extensive mats and are among the main primary producers influencing mineral cycling and primary colonization and development of soils (Elster, 2002). Such mats are present in both the Arctic (Sheath *et al*., 1996; Kim *et al*., 2008, 2011; Pichrtová *et al*., 2016) and Antarctic (Hawes, 1989; Davey, 1991; Skácelová *et al*., 2013).

These polar hydro-terrestrial algal mats are often formed by algae of the genus *Zygnema* C. Agardh that are very well adapted to the harsh conditions of the polar climate, as demonstrated by various ecophysiological studies (Hawes, 1990; Holzinger *et al*., 2009; Pichrtová *et al*., 2013, 2014*a*, 2014*b*; Vilumbrales *et al*., 2013; Choi *et al*., 2015). Nevertheless, both floristic and ecophysiological publications refer only to ‘*Zygnema* spp.’ because species diversity within these mats remains unexplored. Consequently, comparison of results across different studies is practically impossible. Moreover, the lack of knowledge of species diversity precludes the testing of any ecological or biogeographic hypotheses.

The main reason for genus level identification of *Zygnema* specimens is the fact that traditional taxonomy of the genus is based on morphological characteristics connected with sexual reproduction (conjugation), such as wall colour and ornamentation of zygospores or sporangial shape (Kadlubowska, 1984; Stancheva *et al*., 2012). Besides zygospores, other specialized cell types are known in some species of *Zygnema*: parthenospores that result from incomplete conjugation, aplanospores that are formed inside vegetative cells and akinetes that develop directly from vegetative cells by thickening of the cell wall (Kadlubowska, 1984; Stancheva *et al*., 2012). Structure, ornamentation and colouration of such cells is important for definition of some asexual species (Kadlubowska, 1984; Stancheva *et al*., 2012, 2014). Conjugation usually takes place only occasionally. In extreme environmental conditions conjugation is rare or never occurs, which is usually attributed to a trade-off between sexual reproduction and growth (Holzinger *et al*., 2009), a phenomenon widespread in vascular plants (Eckert, 2002). With the exception of *Zygnema* cf. *leiospermum* De Bary from Ellesmere Island in Canada (Elster *et al*., 1997), zygospores of *Zygnema* have not been reported from polar regions.

The situation regarding species identification is different for closely related unicellular desmids, in which species are traditionally defined by vegetative morphological characteristics such as shape, cell wall ornamentation, number of pyrenoids and other features (Coesel & Meesters, 2007). However, modern molecular phylogenetic studies repeatedly show that traditional taxonomy does not reflect the actual relationships, and reveal the existence of cryptic species (Gontcharov, 2008). For example, cryptic lineages that occur sympatrically across Europe were identified within the morphologically well-defined species *Micrasterias truncata* (Corda) ex Bréb (Nemjová *et al*., 2011).

The application of molecular phylogenetic methods is thus also essential for obtaining insight into the diversity of sterile populations of filamentous Zygnematophyceae. The only study investigating molecular phylogeny of the genus *Zygnema* was based on *rbc*L and *cox*1 genes and showed that the genus is split into two major clades, representing distinct differences in reproductive morphology, and that existing taxonomic concepts are not consistent with phylogeny (Stancheva *et al*., 2012). Molecular methods have also been used in two recent experimental studies of polar *Zygnema* strains. The results showed sufficient variability in *rbc*L gene sequences among strains isolated from Svalbard and Antarctica (Pichrtová *et al*., 2013, 2014*b*).

In some other algal groups from polar hydro-terrestrial or terrestrial mats, the application of molecular methods has already provided insight into their diversity and biogeography. *Prasiola crispa* (Lightfoot) Kützing (Trebouxiophyceae, Chlorophyta) is very common in both the Arctic and Antarctic. Recent molecular investigations showed that the Antarctic morphospecies *Prasiola crispa,* in fact, comprises three cryptic species (Moniz *et al*., 2012). Moreover, a similar study in the Arctic even revealed cryptic genera among Prasiolales (Heesch *et al*., 2016). Richter *et al*. (2016) reported that all studied populations of *P. crispa* from a region of Svalbard belong to a single clade (based on *rbc*L phylogeny), although their data also indicated diversification on a population level. In Antarctic Tribonemataceae (Stramenopiles), phylogenetically distant, yet ecologically and morphologically similar filamentous freshwater algae, cryptic genera were also revealed (Rybalka *et al*., 2009), and no biogeographic limitation of individual clades and endemism was revealed (Rybalka *et al*., 2009). By contrast, most lineages of the filamentous green algal genus *Klebsormidium* (Streptophyta) have a geographically limited distribution, and only one cosmopolitan lineage comprises both Arctic and Antarctic isolates (Ryšánek *et al*., 2016).

In the present study, we explored the genetic (*rbc*L sequences) and morphological (width of filaments and chloroplast shape) diversity of *Zygnema* mats from different localities on Svalbard. Key questions were how many different *Zygnema* genotypes could be found within a small sampling region in central Svalbard, and whether individual mats consist of single or multiple genotypes. Additionally, we wondered whether polar *Zygnema* strains form a monophyletic clade, or are closely related to non-polar strains. We searched for sexual reproduction and zygospore formation to identify species, and for vegetative morphological features that could potentially be used to distinguish individual genotypes.

## Materials and methods

### Origin and cultivation of strains

**Fig. 1.**
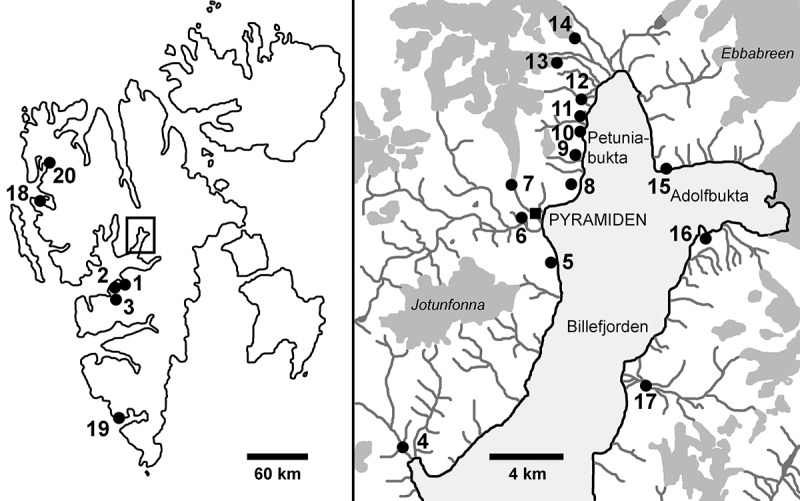
Map showing the locations of original sampling sites of strains investigated in this study. Numbers correspond with Table 1. Dark grey areas indicate glaciers.

Hydro-terrestrial algal mats were sampled at 18 different locations in Central Spitzbergen (1–17) and close to Ny Ålesund (18; Svalbard archipelago; Fig. 1). Sampling sites were shallow pools or slow-running streams supplied by permafrost or snow meltwater. At most locations, several independent mats (39 in total) covering a surface area ranging from several square decimetres to several square metres were sampled. In addition, during 2015 sampling, temperature, pH and conductivity were measured using a portable meter WTW pH/Cond 340i. The natural samples were transported to the Department of Botany, Charles University, using a cooling box. From each mat several individual filaments were isolated and cultured (Table 1). To cover the largest possible area, other available *Zygnema* strains from Svalbard were also included, namely, strains 353-10, 354-10, 355-10, 356-10, 357-10 and 358-10 from the CCCryo culture collection isolated in Hornsund (19) by Thomas Leya in 2010. Additionally, three Antarctic *Zygnema* strains were also investigated, namely CCCryo 280-06, CCCryo 279-06 and the strain MB1 (courtesy of Miloš Barták). All cultures were grown in liquid BBM (Bischoff & Bold, 1963) and maintained at 15°C with continuous light of 35 µmol photons m^–2^ s^–1^. In addition, we tried to induce conjugation by slow desiccation on agar plates, nitrogen starvation and cultivation in a crossed gradient of light and temperature.

**Table 1.** Complete list of sampling localities and number of cultures isolated from each mat and genotype

Locality				Genotype												
No. on map	description	mat	GPS (if available)	B	G	S	L	R	J	A	O	M	V	U	P	N
1	Longyearbyen	1	78°13.143′N, 15°35.28′E	–	–	–	–	–	–	–	–	–	–	7	–	–
		2	78°13.153′N, 15°35.088′E	–	–	–	–	–	–	–	–	–	4	–	–	–
		3		–	–	2	–	–	–	–	–	–	–	–	–	1
2	Björndalen	1	78°13.585′N, 15°19.393′E	–	–	2	–	–	–	–	–	–	–	–	–	–
		2	78°13.071′N, 15°19.664′E	1	–	–	–	–	–	–	1	–	–	–	–	–
		3	78°12.957′N, 15°20.003′E	–	–	2	–	–	–	–	–	–	–	–	–	–
3	Colesdalen	1		–	–	–	–	–	–	–	2	–	–	–	–	–
4	Skansbukta	1		1	–	–	–	–	–	–	–	–	–	–	–	–
5	Garmaksla	1		3	–	–	–	–	–	–	–	–	–	–	–	–
6	Pyramiden	1		4	–	–	–	–	–	–	–	–	–	–	–	–
		2		2	–	–	–	–	–	–	–	–	–	–	–	–
		3		–	–	–	1	–	–	–	–	–	–	–	–	–
		4		–	–	–	–	–	–	3	–	–	–	–	–	–
		5		1	–	1	–	–	1	–	–	–	–	–	–	–
7	Bertilbreen moraine	1		–	–	–	2	–	–	–	–	–	–	–	–	–
8	path to Pyramiden	1	78°39.96′N, 16°25.6333′E	–	1	–	–	–	–	–	–	–	–	–	2	–
9	Petuniahytta	1	78°40.842′N, 16°27.487′E	13	–	8	3	1	–	–	–	–	–	–	–	–
		2	78°40.84′N, 16°27.512′E	–	1	–	1	–	–	6	–	–	–	–	–	–
		3	78°40.838′N, 16°27.523′E	–	1	1	–	–	–	–	–	–	–	–	–	–
		4		3	–	–	3	–	–	–	–	–	–	–	–	–
		5		–	1	–	–	–	–	4	–	–	–	–	–	–
		6		2	–	–	–	–	–	–	–	–	–	–	–	–
10	Old path in Petuniabukta	1	78°41.35′N, 16°26.818′E	–	5	–	–	–	–	–	–	–	–	–	–	–
		2		1	–	–	–	–	–	–	–	–	–	–	–	–
11	Automatic weather station	1	78°42.11′N, 16°27.64′E	4	–	–	–	–	–	–	–	–	–	–	–	–
12	Open top chambers	1		–	2	–	–	–	–	–	–	–	–	–	–	–
		2		–	–	–	–	4	–	–	–	–	–	–	–	–
13	Svenbreen moraine	1		1	–	–	–	1	–	–	–	–	–	–	–	–
14	Hørbyebreen moraine	1		–	2	–	–	–	–	–	–	–	–	–	–	–
15	Fortet	1		–	3	–	–	–	–	–	–	–	–	–	–	–
16	Brucebyen	1		–	–	–	–	5	1	–	–	–	–	–	–	–
17	Mathiesondalen	1		–	1	–	1	–	–	–	–	–	–	–	–	–
		2		–	–	–	–	–	–	–	–	2	–	–	–	–
		3		3	–	–	–	–	1	–	–	–	–	–	–	–
		4		–	1	–	2	–	–	–	–	1	–	–	–	–
		5		–	–	–	–	–	2	–	–	–	–	–	–	–
18	Blomstrand Island	1	78°57.816’N; 12°2.964’E	1	–	–	–	–	–	–	–	–	–	–	–	–
19	Hornsund^a^	1		–	–	6	–	–	–	–	–	–	–	–	–	–
20	Möllerhafen^b^	1		1	–	–	–	–	–	–	–	–	–	–	–	–
	**Number of mats where the genotype was present**			**15**	**10**	**7**	**7**	**4**	**4**	**3**	**2**	**2**	**1**	**1**	**1**	**1**
	Total number of isolated strains per genotype			41	18	22	13	11	5	13	3	3	4	7	2	1

^a^ Cultures obtained from the CCCryo collection.

^b^ Strain sequenced by Gontcharov *et al*. (2004).

## DNA isolation, PCR and phylogenetic analyses

DNA was isolated from vegetative filaments in log-phase of growth according to the protocol of Ryšánek *et al*. (2015), but with 50 µl of Instagene Matrix instead of 100 µl (Bio-Rad Laboratories, USA). Primers RH1 and 1385R (McCourt *et al*., 2000), and newly designed primers ZygF (5’TATGTCAACCACAAAC3’) and ZygR (5’GTATCAAATTCAAATTTA3’), were used for amplification of the *rbc*L gene in reactions containing 13.9 µl of sterile Milli-Q water, 2 µl of MgCl_2_ (25 mM), 2 µl of AmpliTaq Gold 360 Buffer (Applied Biosystems, Carlsbad, California, USA), 0.4 µl of dNTP mix (10 mM), 0.25 µl of forward and reverse primer (25 pmol ml^–1^), 0.2 µl of AmpliTaq Gold 360 DNA Polymerase and 1 µl of DNA (10 ng µl^–1^). Cycling was performed with an initial denaturation for 10 min at 95°C, followed by 35 amplification cycles of 1 min denaturation at 94°C, 1 min annealing at 48°C and 2.5 min extension at 72°C, and a final extension at 72°C for 10 min. Purification of PCR products and DNA sequencing were performed by Macrogen Inc. (Seoul, South Korea). The obtained unique sequences were assigned capital letters A, M, N, O, P, R, S, U and V, and submitted to GenBank under accession numbers MG818336 to MG818344. Capital letters were assigned to individual genotypes based on our recent publications on polar *Zygnema* spp. (Kaplan *et al*., 2013; Pichrtová *et al*., 2013, 2014*b*).

A 1290 nucleotide alignment of 63 sequences (Supplementary material 1) was created using nine of the obtained sequences, our previously published Arctic and Antarctic *Zygnema* spp. and *Zygnemopsis* sp. sequences (Pichrtová *et al*., 2013, 2014*b*), other unique *rbc*L *Zygnema* and *Zygnemopsis* sequences available in the GenBank database and several closely related Zygnematophyceae sequences (based on BLAST searches and Hall *et al*. 2008). The alignment also included Arctic *Zygnema* strain CCAC 1384B (ASW 07067), which was isolated from Möllerhafen in 1992 and sequenced by Gontcharov *et al*. (2004). This strain is included in the map and the list of sampling sites (no. 20; Fig. 1; Table 1). A list of all strains used in the final phylogenetic tree and their GenBank accession numbers can be found in the Supplementary material 2.

Three different phylogenetic analyses were performed: Bayesian inference (BI), maximum likelihood (ML) and weighted parsimony (wMP, character-weighted). Sequence evolution models were determined as GTR+gamma for the first codon position, JC+I for the second position and GTR+I+gamma for the third position using MrModel Test 2.3 (Nylander, 2004) with the Akaike Information Criterion. The BI phylogenetic tree was constructed using MrBayes 3.2.6 (Ronquist & Huelsenbeck, 2003). Two parallel Markov chain Monte Carlo runs were carried out for 3 000 000 generations, each with one cold and three heated chains. Convergence of the two cold chains was checked by the average standard deviation of split frequencies, and the value was 0.003835. Trees and parameters were sampled every 100 generations, and trees from the initial 1000 generations were discarded using the sumt burnin function. Bootstrap analysis was performed by ML in Garli 2.0 (Zwickl, 2006) and PAUP* Portable version 4.0b 10 (Swofford, 2002). ML analyses consisted of rapid heuristic searches (100 pseudoreplicates) using automatic termination (genthreshfortopoterm command set to 100 000). The wMP bootstrapping was performed using heuristic searches with 100 random sequence addition replicates, tree bisection reconnection swapping and random addition of sequences (number limited to 10 000 for each replicate). The Bayesian tree was midpoint-rooted and further processed using Mega 6 (Tamura *et al*., 2013) and Adobe Illustrator CS3 (Adobe Systems, San Jose, California, USA).

### 

#### Light microscopy and cell measurements

Three-week-old cultures were used for light microscopy observations using a Zeiss Axiovert 200M microscope equipped with a 63×1.4 NA objective lens (Carl Zeiss Microscopy GmbH, Jena, Germany). Images were captured with an Axiocam MRc5 camera and Zeiss Axiovision software. Sixty cells for each genotype were randomly chosen for width measurements in ImageJ 1.50i software (http://imagej.nih.gov/ij). Where possible, two or three strains per genotype were used, in which case 30 cells per strain were measured (20 respectively).

Levene’s test for homogeneity of variance confirmed that variation in cell width among genotypes was not homogenous (*F* = 22.70333, *p* < 0.0001). Therefore, differences in cell width among individual genotypes were tested by non-parametric Kruskal–Wallis tests and subsequent Mann–Whitney pairwise comparisons with Bonferroni correction using PAST 2.17c (Hammer *et al*., 2001).

Conjugation and zygospore formation were recorded in a natural sample. The sample was stored in the habitat water diluted with distilled water 50:50 and incubated under optimal growth conditions, 17°C and continuous light. Morphological variability during conjugation and all zygospore developmental stages were continuously monitored and photodocumented using an Olympus BX51 light microscope (Nomarski differential contrast, phase contrast) with Olympus Camedia C-5060Z digital microphotographic equipment (Olympus, Tokyo, Japan). Portions of samples were fixed with 1.5% glutaraldehyde for later determination, and portions of mature samples were treated with 10% KOH to distinguish spore wall layers (Stancheva *et al*., 2012). After completion of zygospore germination, the species was determined using the traditional literature (Transeau, 1951; Randhawa, 1959; Gauthier-Lièvre, 1965; Kadlubowska, 1984).

#### Confocal laser scanning microscopy

Chloroplast morphology was investigated using a Leica TCS SP2 laser scanning confocal microscope (Leica Microsystems, Wetzlar, Germany) equipped with an Argon-Krypton laser. Several *Zygnema* filaments were transferred to a drop of distilled water and mounted together with a piece of solidified agar medium to prevent movement during scanning. A 488 nm excitation wavelength and an AOBS ﬁlter-free system (Leica Microsystems) collecting emitted light between 498 and 700 nm were used. A Leica DM IRE2 inverted microscope (Leica Microsystems) was used to visualize a series of chloroplast optical sections for three-dimensional (3D) morphology reconstruction. Chlorophyll autoﬂuorescence was exploited for visualization of chloroplast structure, and chloroplast 3D morphology reconstructions were produced using ImageJ version 1.50 with the Fiji image processing package (Schindelin *et al*., 2012).

### Transmission electron microscopy (TEM)

Transmission electron microscopy (TEM) was essentially performed as previously described (Holzinger *et al*., 2009). The entire fixation and embedding procedure was performed immediately in Svalbard. Freshly harvested samples were fixed in 2.5% glutaraldehyde in 50 mM cacodylate buffer for 2 h, washed in the same buffer, then embedded in 3% agarose. Samples were then post-fixed in 1% OsO_4_ at 4°C for 12 h, followed by dehydration using increasing ethanol concentrations, transferred to propylene oxide, embedded in modified Spurr’s embedding resin and heat polymerized. Samples were further processed in the laboratory in Innsbruck, and ultrathin sections were prepared with a Leica Ultracut microtome, and counterstained with uranyl acetate and Reynold’s lead citrate. Sections were viewed using a Zeiss Libra 120 TEM instrument at 80 kV, and images were captured with a TRS 2k SSCCD camera and further processed with Adobe Photoshop Elements 11 software (Adobe Systems, San Jose, California, USA).

## Results

### Molecular diversity and occurrence of *Zygnema* mats

**Fig. 2.**
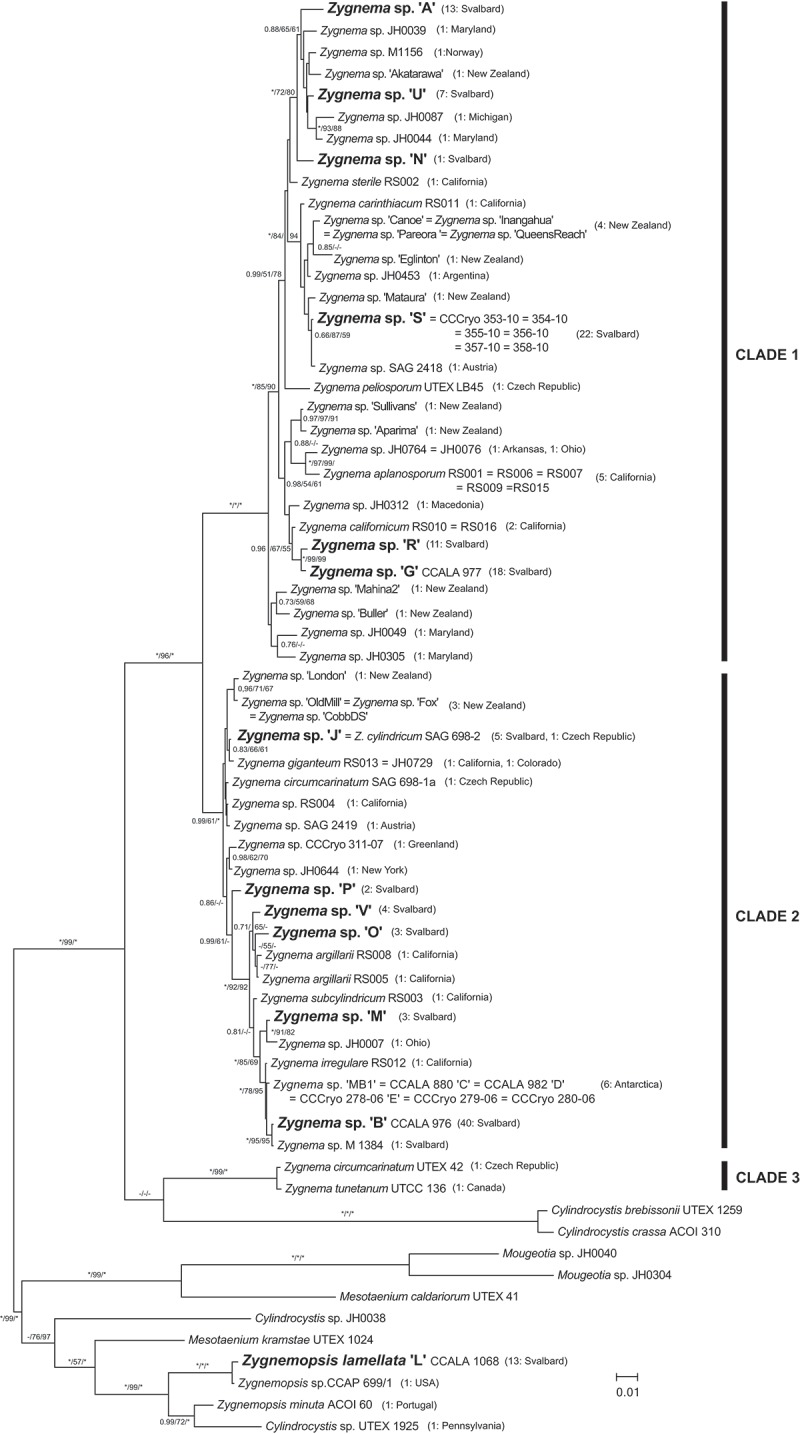
Phylogenetic tree of genera *Zygnema, Zygnemopsis* and other closely related Zygnematophyceae. A midpoint-rooted Bayesian tree of *rbc*L sequences is shown. Genotypes from Svalbard are in bold. Values at branches indicate Bayesian posterior probabilities (BI PP), maximum likelihood (ML) and maximum parsimony (MP) bootstrap values (BS). Asterisks indicate BI PP = 1.00, and ML and MP BS = 100; dashes indicate BI PP <0.8 and ML and MP BS <50. The number of strains isolated for each *Zygnema*/*Zygnemopsis* genotype and their geographic origin are given in parentheses.

Chloroplast-encoded *rbc*L sequences were obtained for 143 Arctic strains with vegetative *Zygnema*-like morphology isolated from 39 different mats at 20 different localities on Svalbard (Fig. 1; Table 1). Phylogenetic analyses revealed 12 different *Zygnema* genotypes (A, B, G, J, M, N, O, P, R, S, U and V) from the Arctic and one member of the genus *Zygnemopsis* (L; Fig. 2). The identified genotypes were evenly distributed among genotypes of non-polar origin in the two main clades of the genus, and did not form any monophyletic clusters (Fig. 2). Only genotypes G and R, which differed by 5 bp within the investigated region, formed a separate well-supported lineage (Fig. 2). Some of the Arctic genotypes were very closely related to others, such as *Zygnema* sp. S that differed only at one site (without altering the translation product) from the Austrian strain *Zygnema* sp. SAG 2418. An Arctic genotype J was even identical to previously described *Z. cylindricum* from the Czech Republic (strain SAG 698-2). The genus *Zygnemopsis* formed a monophyletic clade only distantly related to the genus *Zygnema*, but closely related to another filamentous genus (*Mougeotia*).

Additionally, we also investigated all Antarctic *Zygnema* strains currently available, consisting of three newly sequenced strains (CCCryo 280-06, 279-06 and MB1) and published sequences of strains *Zygnema* sp. C, D and E (Kaplan *et al*., 2013; Pichrtová *et al*., 2013). All shared an identical *rbc*L sequence, and were closely related to other strains: Their *rbc*L sequence differed by only 1 bp from *Zygnema irregulare* Krieger isolated in California (strain RS012, Stancheva *et al*., 2012) and by 4 bp from the most common Arctic genotype B. Nevertheless, all these sequences encode an identical translation product.

The frequency of individual genotypes differed; four genotypes were found at a single site only, whereas genotype B was the most common (present in 41 out of 143 strains, and in 15 out of 39 mats; Table 1). Notably, the only *rbc*L sequence of Svalbard *Zygnema* obtained from public databases, strain M1384 isolated in Möllerhafen, is virtually identical to our B genotype (*rbc*L sequences differ at only one site, where M1384 has Y instead of T); hence both are referred to as genotype B in Table 1.

Surprisingly, a certain level of diversity was revealed within individual mats, which were presumed to be formed by a single species. Fourteen out of 39 mats included more than one genotype. One site, mat no. 1 at locality 9 Petuniahytta (Elster & Rachlewicz, 2012) with an area of 1 m^2^, was selected for more detailed investigation. We isolated and sequenced 25 *Zygnema* strains from this location, revealing four different genotypes (Table 1).

Environmental parameters are available for several mats sampled in August 2015. Water temperature did not exceed 12°C in most cases, pH was neutral to alkaline and conductivity ranged between 37 and 880 µS cm^–1^ (Supplementary material 3).

### Sexual reproduction and zygospore formation

**Figs 3–9.**
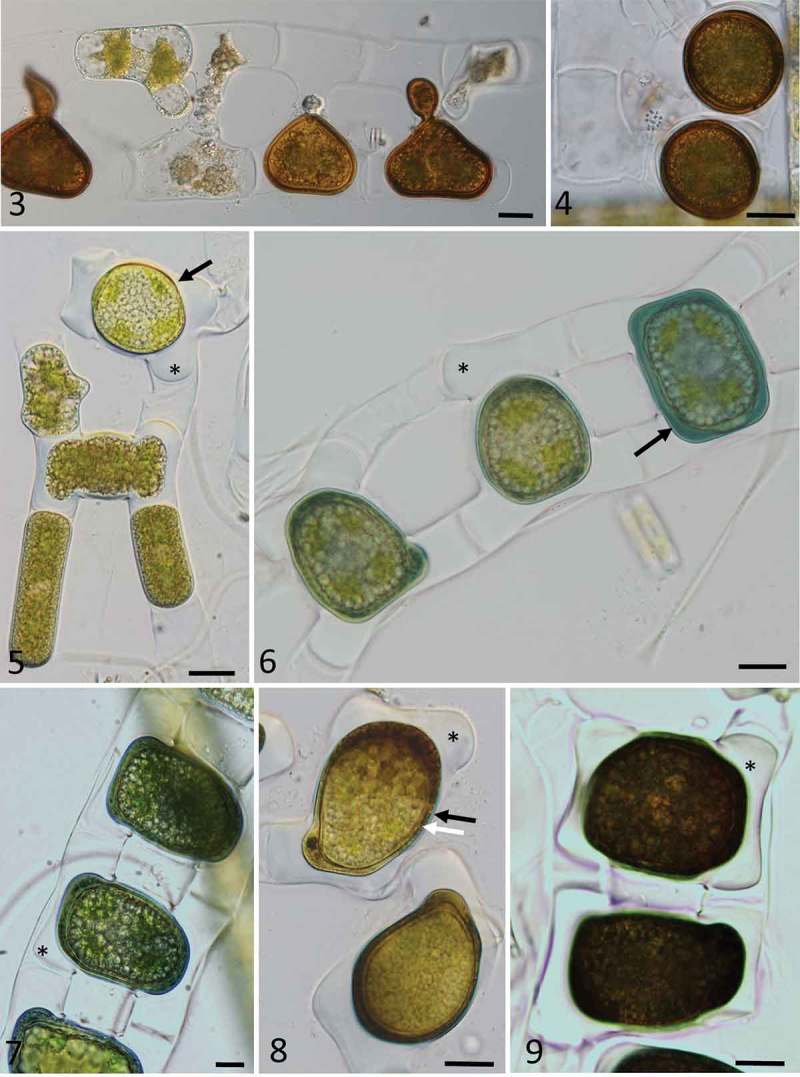
Light microscopy images of zygospores of *Zygnema* cf. *calosporum* (Figs 3, 4) and *Zygnemopsis lamellata* (Figs 5–9) collected in Svalbard. Fig. 3. Conjugating filaments, with partially developed zygospores; Fig. 4. mature zygospores; Fig. 5. conjugating stage (arrow) and fully fused zygospore (arrow) with massive appendages (asterisk); Fig. 6. zygospores with blue mesospore (black arrow) at different stages of development; Fig. 7. zygospores still in conjugating filaments, positioned in the middle, with appendages (asterisk); Fig. 8. zygospores with a two-layered mesospore clearly visible, comprising an outer layer in blue (black arrow), and an inner layer scrobiculate (white arrow), with appendages marked by an asterisk; Fig. 9. dark appearance of fully developed zygospores, showing appendages of the exospore (asterisk); Scale bars: Figs 3–9 = 20 µm.

We did not observe formation of asexual spores (aplanospores, akinetes) in this study, sexual reproduction was observed only twice. First, we observed conjugation and formation of zygospores of *Zygnema* in material collected in 2013 at the Garmaksla sampling site (no. 5; Figs 3, 4). Unfortunately, this sample was small and did not provide sufficient information for species determination, nor were we able to establish a culture from conjugating filaments for DNA analysis. Based on the few images without the final stage of cell wall development, the zygospores best resemble *Z. calosporum* C.-C. Jao. This species was originally described from China, with spherical or almost spherical brown zygospores from 29×35 to 32×38 μm in size and with filament width range 20–26 mm.

**Figs 10–14.**
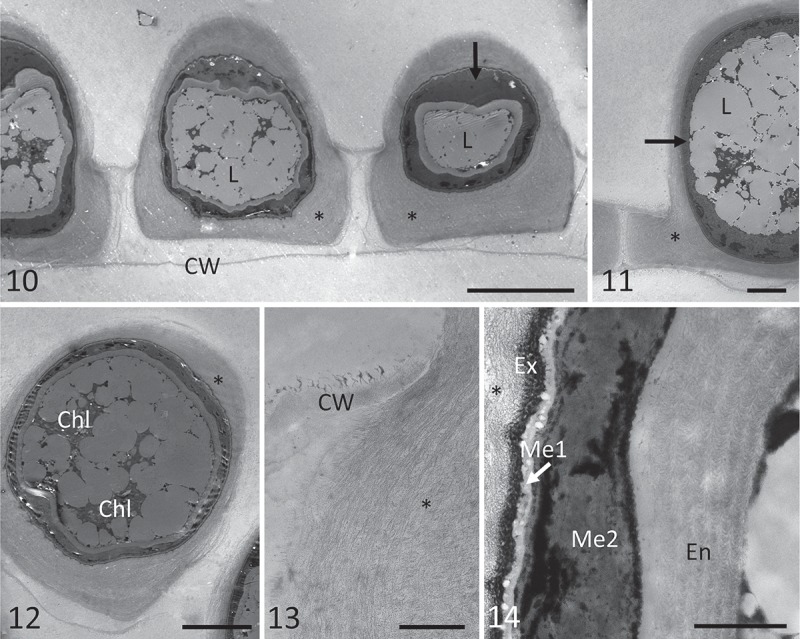
Transmission electron micrographs of zygospores of *Zygnemopsis lamellata* collected in the field. Fig. 10. Several zygospores still in the original filaments, showing an electron-dense inner mesospore layer (arrow), and exospore surrounded by pectic-cellulosic appendages (‘wings’) marked with an asterisk; Fig. 11. electron-dense mesospore layer (arrow), with lipid bodies inside the zygospores, and appendages marked by an asterisk; Fig. 12. chloroplasts in zygospores; Fig. 13. pectic-cellulosic appendage within the gametangium; Fig. 14. exospore (Ex), two-layered mesospore (Me1 = electron-translucent, Me2 = electron-dense and irregular) and highly sculptured endospore (En). Abbreviations are Chl = chloroplast, CW = cell wall of the mother cell, Ex = exospore, L = lipids, Me1 = outer mesospore layer, Me2 = inner mesospore layer, En = endospore. Scale bars: Fig. 1 = 20 µm; Figs 11–12 = 10 µm; Fig. 13 = 2 µm; Fig. 14 = 1 µm.

In 2015, massive conjugation occurred in a single mat (mat 4) from the Petuniahytta sampling site (no. 9). The water at the sampling site had neutral pH and conductivity 409 µS cm^–1^ (Supplementary material 3). Field material was collected and development of zygospores was successfully tracked in laboratory conditions (Figs 5–9). Scalariform conjugation was observed exclusively, and zygospores were generally quadrangular pillow-shaped, quadrately ovoid, and laterally compressed (Figs 5, 6), and only rarely globose. An early zygospore was formed in the conjugation tube, which was filled, mostly protruding to both gametangia. The zygospore was already surrounded by a lamellate wall (exospore) after zygospore formation, and this was surrounded by a pectic-cellulosic appendage with a wing-like shape (Fig. 5, asterisk). The original filaments often fell apart soon afterwards. A two-layered mesospore developed as a colourless wall that turned blue-green very quickly (Figs 6, 7). The inner mesospore layer evolved slowly into a yellow-brown layer with scrobiculate ornamentation (Fig. 8). The external layer became thinner and undulate, often poorly visible, but still blue (Fig. 8). In the final stage, the blue-coloured layer was generally very thin or completely absent, and zygospores became darker (Fig. 9). Based on these morphological features, the species was identified as *Zygnemopsis lamellata* Randhawa. The original description comprises zygospores with 44–52 μm in diameter, the same pattern of their formation and appearance and vegetative filaments ranging 15–21 μm in width. In transmission electron micrographs, zygospores at different stages of development were observed and characterized from field material that was fixed immediately for TEM analysis at the collection site on Svalbard. The cell lumina of zygospores contained huge quantities of lipids, and chloroplasts occupied only a small area (Figs 10, 11). Zygospores remained in the central parts of the copulation channel (Fig. 10), and due to the orientation of the sectioning, only one gametangium (original filament) was visible. Gametangia were almost entirely filled by a pectic-cellulosic appendage that covered the exospore (Figs 10, 12). Within the appendage, the loose arrangement of cellulose fibrils was clearly visible (Fig. 13). The structure of the ‘mother cell wall’ of gametangia was clearly distinct from these layers (Fig. 14), and zygospores were surrounded by an exospore and two distinct mesospore layers. The outer mesospore layer (Me1, Fig. 14) was electron-translucent and thin, while the inner mesospore layer (Me2) was irregular and electron-dense, indicating differences in chemical composition. The endospore was of intermediate electron density, and composed of several highly symmetrically arranged fibrillary layers (Fig. 14).

### Vegetative morphology

The genera *Zygnema* and *Zygnemopsis* share a very similar vegetative morphology, even though they are not closely related. Using light microscopy, they can be easily confused (for *Zygnema* genotypes, see Figs 15–26; for *Zygnemopsis lamellata*, Fig. 27). However, when the morphology of the chloroplast was observed using confocal laser scanning microscopy, the differences were quite clear (Figs 28–39 for *Zygnema* spp., Fig. 40 for *Zygnemopsis*). *Zygnemopsis* possesses two compact lobate chloroplasts per cell (Fig. 40), with the nucleus placed between chloroplasts in the middle of the cell. The terminal lobes are wide, flat and rounded in shape, with relatively deep incisions. Chloroplasts occupy most of the cell’s interior, and they are porous or spongy inside (not shown), with small, irregular cavities on the surface that give a granular appearance. The flat lobes are often appressed to the cell wall, and this feature seems to be important for distinguishing *Zygnemopsis* from *Zygnema*.

**Figs 15–27.**
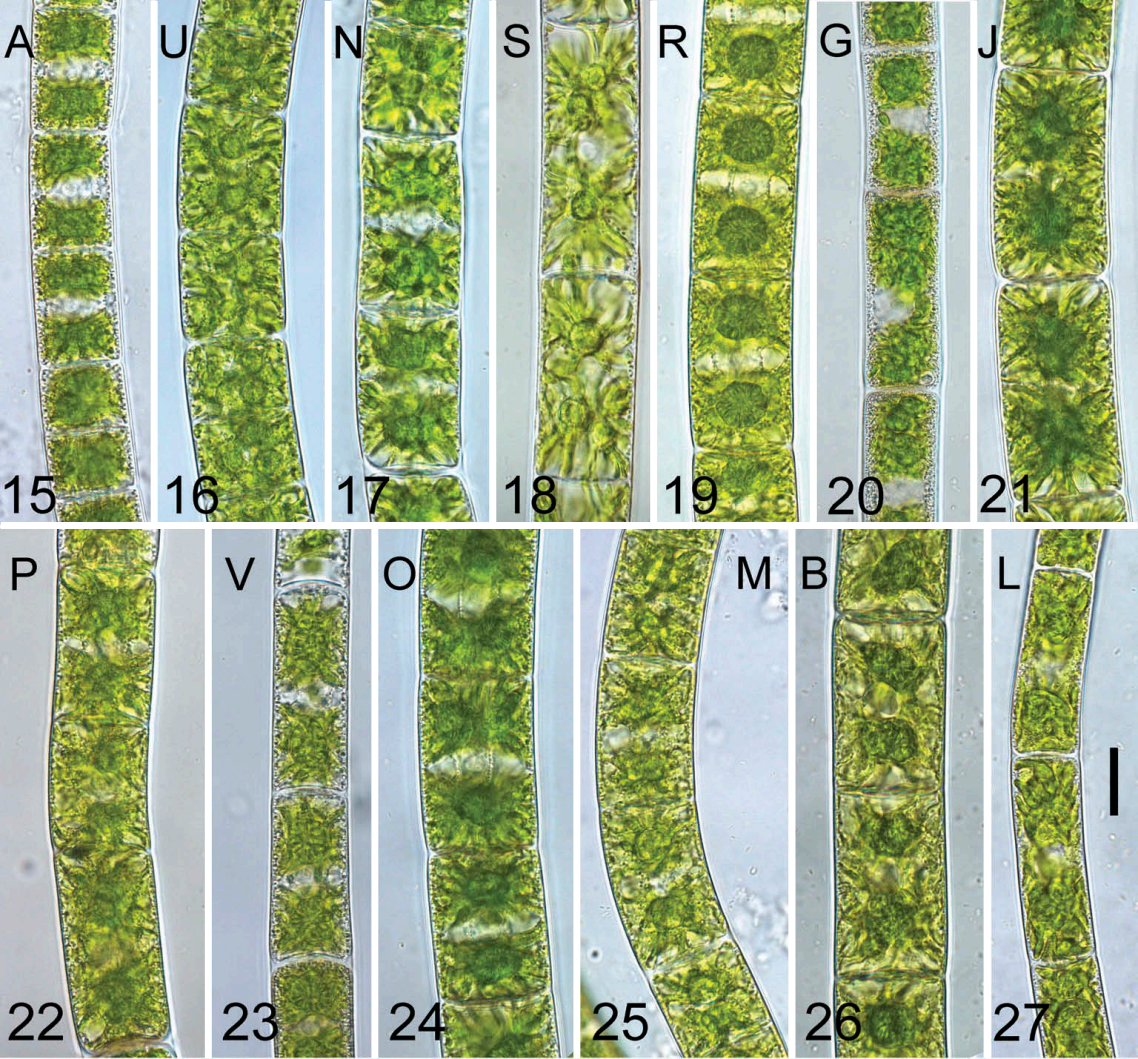
Young vegetative cells (3 weeks after transfer to fresh medium) of the investigated genotypes. Fig. 15. Genotype A; Fig. 16., U; Fig. 17. N; Fig. 18. S; Fig. 19. R; Fig. 20. G; Fig. 21. J; Fig. 22. P; Fig. 23. V; Fig. 24. O; Fig. 25. M; Fig. 26. B; Fig. 27. L. Scale bar = 20 µm in all images. Genotypes are ordered according to phylogeny described in Fig. 2.

**Figs 28–40.**
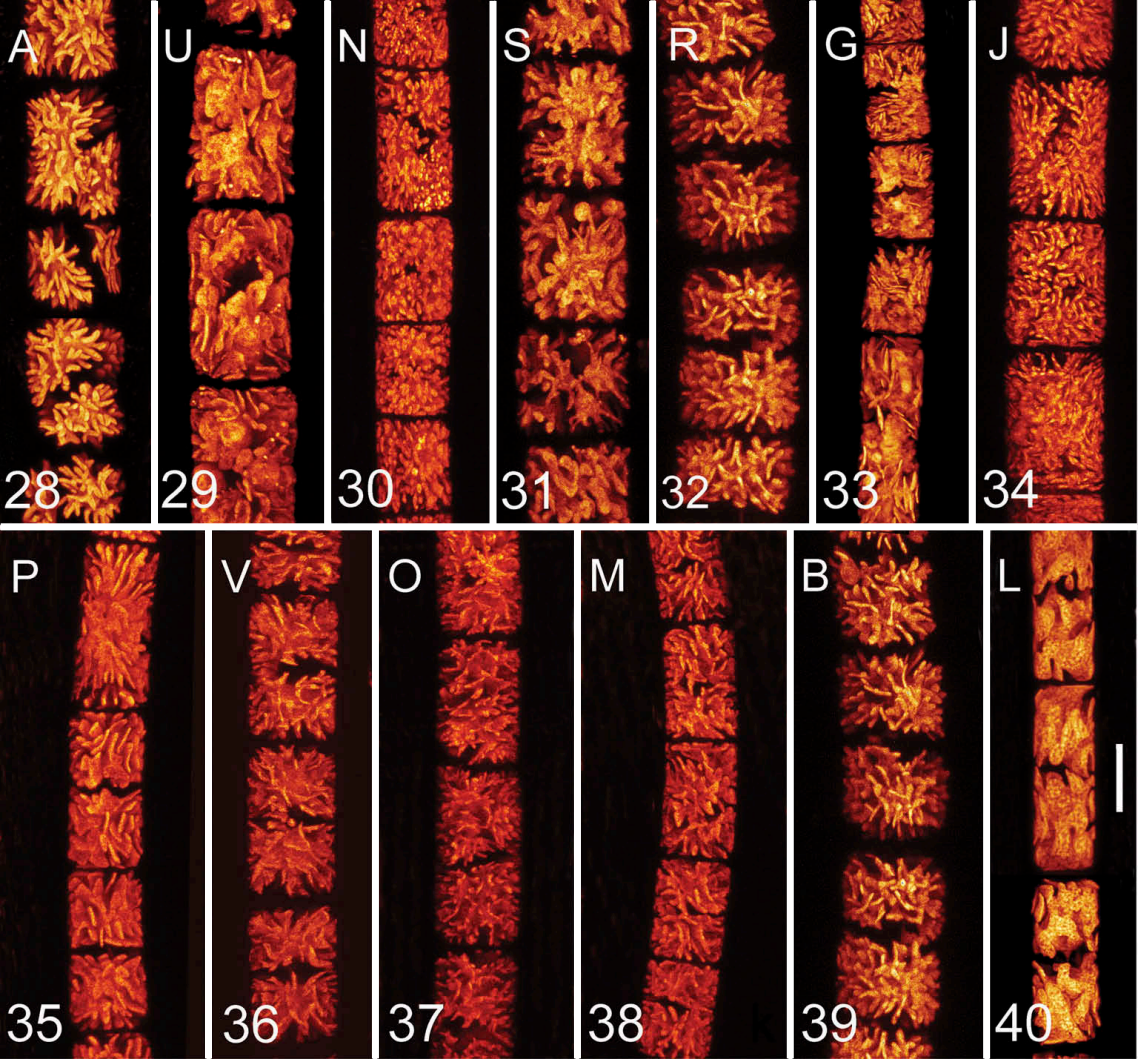
Confocal laser scanning microscopy of young vegetative cells of the investigated genotypes. Fig. 28. Genotype A; Fig. 29. U; Fig. 30. N; Fig. 31. S; Fig. 32. R; Fig. 33. G; Fig. 34. J; Fig. 35. P; Fig. 36. V; Fig. 37. O; Fig. 38. M; Fig. 39. B; Fig. 40. L. Scale bar = 20 µm in all images. Genotypes are ordered according to phylogeny described in Fig. 2.

Confocal laser scanning microscopy was also applied to characterize individual genotypes of *Zygnema* based on the morphology of their star-shaped chloroplasts. When observed in exponentially growing cultures, chloroplast shape remained stable. Generally, within the studied *Zygnema* strains, four groups of plastids were recognized: (i) chloroplasts with very delicate and thin lobules as shown for genotypes N (Fig. 30), J (Fig. 34), V (Fig. 36), O (Fig. 37) and M (Fig. 38); (ii) chloroplasts with lobes wider at the base and terminated by a blunt tip as shown for A (Fig. 28), R (Fig. 32) and B (Fig. 39); (iii) chloroplasts with flat, wide terminal lobes as shown for U (Fig. 29) and S (Fig. 31); (iv) chloroplasts with prolonged lamellate lobes as shown for G (Fig. 33) and P (Fig. 35). However, chloroplast shape does not appear to reflect the phylogenetic position of the strains. Unrelated genotypes may possess a similar chloroplast type and, conversely, genotypes from one lineage (e.g. R and G; Figs 32, 33) can differ considerably in chloroplast morphology.

**Fig. 41.**
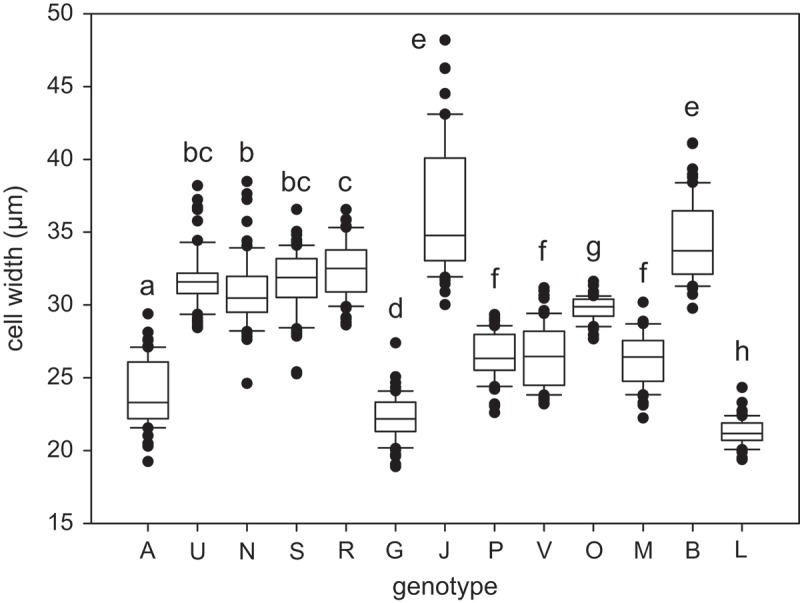
Cell width of all investigated genotypes (*n* = 60). The line within the box marks the median, boundaries indicate the 25^th^ and 75^th^ percentiles, error bars indicate the 10^th^ and 90^th^ percentiles, and individual points denote outliers (samples with values outside this range). Genotypes that do not significantly differ from each other share at least one letter, while those significantly different from each other do not share any letters (*p* < 0.05; Kruskal–Wallis test with multiple comparisons).

In addition to chloroplast shape, we also characterized genotypes according to filament width, and mean cell width differed significantly among nearly all genotypes (Kruskal–Wallis test and Mann–Whitney pairwise comparisons with Bonferroni correction; Fig. 41). However, the measured values strongly overlapped among individual genotypes, large differences in cell width variance were observed (Fig. 41) and congruence between filament width and chloroplast morphology was not observed (the results of statistical analyses can be found in Supplementary material 4).

## Discussion

### Diversity of sterile ‘*Zygnema/Zygnemopsis*’ mats on Svalbard

The diversity of conjugating green algae on Svalbard displaying vegetative *Zygnema* morphology was surprisingly high. Phylogenetic analyses based on the *rbc*L gene revealed 12 different *Zygnema* genotypes and one *Zygnemopsis* genotype among 143 strains with almost uniform vegetative appearance. Until recently, such algae collected from Svalbard were always reported only as *Zygnema* sp. (e.g. Kim *et al*., 2008, 2011; Holzinger *et al*., 2009). In this study, we confirmed the occurrence of *Zygnemopsis* (Pichrtová *et al*., 2014*b*), and further determined the species as *Zygnemopsis lamellata* by morphological characterization of a fertile sample observed in the field.

Traditional taxonomy of *Zygnema* and *Zygnemopsis* is based on morphological characteristics connected with sexual reproduction. Consequently, studies describing the diversity of these algae are limited to records of fertile samples (Novis, 2004; Poulíčková *et al*., 2007; Kim *et al*., 2012; Stancheva *et al*., 2012). Novis (2004) reported only one fertile specimen (i.e. one species) of *Zygnema* from New Zealand. Poulíčková *et al*. (2007) reported various zygospores but identified only one species from a sampling site in the Czech Republic. Kim *et al*. (2012) reported two species of *Zygnema* in Korea despite more than 3 years of collecting. Finally, Stancheva *et al*. (2012) reported eight species (including two new to science) from streams in California, one of which was assigned as *Z. sterile* Transeau based on akinetes, because the reproduction of this species is unknown (Transeau 1951). These researchers also reported numerous sterile samples that were not investigated using molecular methods (Stancheva *et al*., 2012).

However, a few recent ecophysiological studies indicated that there is diversity hidden within non-reproducing field populations of *Zygnema* or *Zygnemopsis* (Kaplan *et al*. 2013; Pichrtová *et al*. 2013, 2014*b*). In another filamentous conjugating green algal genus, *Spirogyra*, molecular diversity analysis of sterile strains from Germany and Austria revealed 53 different SSU rDNA genotypes within 130 isolated strains, and the genus was split into eight independent lineages (Chen *et al*., 2012). Similarly, Stancheva *et al*. (2013) characterized, by morphological and molecular methods, 15 *Spirogyra* species from streams in California. In general, application of molecular methods in morphologically uniform protists usually reveals hidden diversity on various levels, depending on the selected marker(s) (e.g. Nemjová *et al*., 2011; Moniz *et al*., 2012; Heesch *et al*., 2016).

Despite many recent studies, the taxonomy and phylogeny of many Zygnematophyceae genera have not yet been revised. It has been repeatedly shown that morphology is not congruent with phylogeny (Gontcharov & Melkonian, 2005; Chen *et al*., 2012; Stancheva *et al*. 2013). Currently, both *Zygnema* and *Zygnemopsis* genera belong to order Zygnematales and family Zygnemataceae (Guiry, 2013). Gontcharov (2008) mentioned 139 species of *Zygnema*, but 212 species are currently listed as taxonomically accepted in AlgaeBase (www.algaebase.org). Nevertheless, many were observed only once, making it highly probable that their ‘unique’ morphology reflects phenotypic plasticity, and that the number of species has been overestimated. To date, only 13 species in public databases have been assigned based on both morphological determination and *rbc*L sequence analysis (Fig. 2). Moreover, due to a lack of taxonomic revision and/or hybridization experiments, the number of base pair changes sufficient for species-level discrimination has not been determined. Therefore, we use the term ‘genotype’ instead of ‘species’. The R and G pair are the most closely related Arctic genotypes, and they differ at five sites, while two well-defined species, *Z. cylindricum* and *Z. giganteum* Randhawa, differ at only two sites.

A recent phylogenetic study of *Zygnema* split the genus into two main well-supported clades (Stancheva *et al*., 2012), which corresponded to the zygospore colour in *Zygnema*, i.e. blue vs. brown. A new understanding of the phylogenetic position of the genus *Zygogonium* was recently proposed based on *atpB, psbC* and *rbc*L genes, as well as morphological features (Stancheva *et al*., 2014). Some of the former *Zygogonium* species were transferred to *Zygnema*, based on combination of chloroplast structure and reproductive features. Thus, our clade 3 (Fig. 2) contains *Zygnema tunetanum*, a species originally described as *Zygogonium tunetanum*. Consistent with this previously published phylogeny, our genotypes were distributed within the two largest clades (Fig. 2). It can be hypothesized that strains A, U, N, S, R and G would produce spores with blue mesospore layer and genotypes J, P, V, O, M and B with yellow or brown (Stancheva *et al*. 2012).

*Zygnemopsis* is a small genus with only 43 described species. It is not a sister lineage to *Zygnema*, since they are only distantly related. There are only four public *rbc*L sequences that form a monophyletic clade, including one *Cylindrocystis* strain, which was most likely incorrectly determined using morphological methods alone.

#### Occurrence of fertile specimens

Although most specimens were sterile, we also observed conjugation and zygospore formation in our field samples, representing the first record of sexual reproduction in *Zygnema* and *Zygnemopsis* from Svalbard. Production of zygospores is energetically demanding, because they develop a three-layered cell wall with a middle layer containing sporopollenin-like material (Poulíčková *et al*., 2007). Moreover, the short vegetation period and water loss of mats might prevent complete development of zygospores.

Based on the morphology of zygospores and gametangia in field samples found at site 4 in 2015, we assigned our *Zygnemopsis* sp. L sample as *Z. lamellata*. Its phylogenetic position has already been determined in our previous work (Pichrtová *et al*., 2014*b*). Zygospores of *Zygnemopsis* were distinguishable from those of *Zygnema* because they have four lamellate and solid, wing-like appendages attached (Transeau, 1951), and the zygospores themselves are quadrangular pillow-shaped, as is the case for *Zygnemopsis lamellata*. We confirmed the presence of wing-like lamellate appendages surrounding the exospore using light microscopy (Figs 5–9), as well as by TEM (Figs 10–14), which clearly showed that these structures are cellulosic-pectic, with a loose fibrillary appearance (Fig. 13). The function of the ‘wings’ remains unclear, although pectic layers are common in Zygnematophyceae, and usually relate to the water-holding capacity, suggesting that the lamellate appendages could be beneficial when water availability is scarce during zygospore formation. *Zygnema*/*Zygnemopsis* mats have been frequently found to dry out during the vegetative season (Holzinger *et al*., 2009; Pichrtová *et al*., 2014*a*), which could result in incomplete zygospore development. The appendages may therefore help to overcome this problem in *Zygnemopsis lamellata*.

Our light microscopy observations of spore layers were consistent with the differences in the electron density of the two mesospore layers observed by TEM: while the outer layer (Me1) is electron-translucent and thin (Fig. 14), the inner layer is electron-dense and irregular, hence the scrobiculate appearance. Endospores exhibited a highly organized fibrillary structure of medium electron density (Fig. 14), consistent with the translucent appearance in light microscopy observations.

Unfortunately, *Zygnema* zygospores from a natural sample collected in 2013 could not be determined precisely, but some features of the zygospore wall indicate that it could possibly be *Zygnema* cf. *calosporum*.

The distribution and the ecological characterization of *Zygnema calosporum* and *Zygnemopsis lamellata* are not sufficiently described. There are some records of *Z. lamellata* from India (Randhawa, 1937) and Norway (Kadlubowska, 1984). *Zygnema calosporum* is known from China, Algeria (Kadlubowska, 1984) and is also reported from the Netherlands (Simons, 1987).

### Biogeographic and ecological patterns in polar microalgae

The 12 Arctic *Zygnema* genotypes investigated in this study cluster into two main clades of the genus (Fig. 2) and are intermixed with, and in some cases closely related to, strains isolated from different regions. The difference between the Antarctic strains and the Californian strain of *Z. irregulare* is so small (1 bp) that they could be considered a single species, and similarities in morphology (formation of akinetes) and stress tolerance support this theory (Fuller, 2013; Pichrtová *et al*., 2014*b*). Similarly, other protist genera comprise cosmopolitan lineages where even Arctic and Antarctic strains cluster together (Heesch *et al*., 2016; Hodač *et al*., 2016; Ryšánek *et al*., 2016).

This genetic similarity is not surprising, since the ubiquity theory, assuming cosmopolitan distribution and unlimited dispersal of protists, was proposed as an explanation for the worldwide distribution of protists (Finlay *et al*., 1996; Finlay, 2002). However, this theory has been tested many times, and numerous examples of protist species with limited distribution are known. Another theory of protist distribution, the moderate endemicity model, was proposed which admits the existence of endemic species (Foissner, 1999, 2006). For example, Antarctic and Arctic endemites are common within diatom species complexes (Souffreau *et al*., 2013; Kociolek *et al*., 2017; Pinseel *et al*., 2017). Antarctic microchlorophytes (De Wever *et al*., 2009) or *Prasiola* (Moniz *et al*., 2012) also possess endemic lineages. Typically, different species within a single protist genus may exhibit contrasting distribution patterns (Heesch *et al*., 2016; Ryšánek *et al*., 2016). Polar *Zygnema* strains do not form any clear endemic lineage, but we cannot exclude the possibility that some of the reported Arctic genotypes are endemic to the Arctic region. However, to support this hypothesis more extensive molecular data from other regions are needed, because even putative endemites can be redetected elsewhere, depending only on sampling effort.

This study was not specifically designed to test ecological differences between regions, but some patterns were nonetheless observed. For example, genotypes U and V were found only in Longyearbyen, and were never observed in Petuniabukta where most samples were taken. The sampling site in Longyearbyen was located high up a mountain beneath a snow field (conductivity 40–60 µS cm^–1^; Supplementary material 3), whereas sites in Petuniabukta were located near the seashore and therefore rich in minerals (Komárek *et al*., 2012). Also, climatic conditions differed between Petuniabukta and Longyearbyen (Láska *et al*., 2012). Ecological conditions may therefore play an important role in determining the distribution and abundance of some genotypes. The lineages of another streptophytic algal genus, *Klebsormidium*, also showed ecological preferences (Škaloud & Rindi, 2013). Similarly, individual species of polar Tribonemataceae were defined ecologically rather than biogeographically (Rybalka *et al*., 2009), and the diversity of *Prasiola* from Svalbard was also correlated with environmental conditions (Richter *et al*., 2016).

### Possible role of seasonal changes in local diversity

Molecular investigations also revealed that 14 out of 39 comparably sized mats were composed of two or more genotypes of *Zygnema* or *Zygnemopsis lamellata* and the mat chosen for a more thorough investigation consisted of four genotypes. The coexistence of different genotypes indicates that they share similar ecological preferences. Similarly, up to three genotypes of *Spirogyra* were detected at a single site (Chen *et al*., 2012; Stancheva *et al*. 2013), and sympatric occurrence of cryptic (or pseudocryptic) species has been described for other microalgal assemblages such as diatoms (Vanormelingen *et al*., 2008) and desmids (Nemjová *et al*., 2011). Nevertheless, it should be considered that our genotypes may not represent true ‘cryptic species’, because we could not use all distinguishing features due to the absence of zygospores. Moreover, the co-occurrence of various well-defined species of a single genus at one site is a relatively common phenomenon in Zygnematophyceae (Nováková, 2002; Štěpánková *et al*., 2012).

Co-occurrence of various genotypes is supported by the annual characteristics of mats, since new biomass rapidly develops every year from a small inoculum, and the time for competition is rather limited (Pichrtová *et al*., 2016). Interestingly, sites that were investigated repeatedly (sites 10, 11 and 15, and mat 2 at site 9; Fig. 1) retained the same genotypes, suggesting that survival of cells from the previous season, together with their rapid spring growth, is more important for colonization of the pool than allochthonous transport of new genotypes from other sites. In the absence of zygospores, the role of surviving cells is played by pre-akinetes, which are hardened, old vegetative cells resistant to various environmental stresses (Holzinger *et al*., 2009; Pichrtová *et al*., 2014*a*, 2014*b*, 2016; Herburger *et al*., 2015).

### Vegetative morphology and indications of polyploidy

The very rare occurrence of zygospores on Svalbard precludes the morphological determination of individual species in field conditions. Moreover, despite the experimental effort, induction of zygospore formation in culture conditions was not successful. Therefore, we decided to test whether our genotypes could be discriminated based solely on their vegetative morphology, even though it is well known that the molecular diversity of filamentous Zygnematophyceae is far greater than indicated by the lack of morphological variation (Chen *et al*., 2012). Despite being based on generative morphological features, traditional species description of filamentous Zygnematophyceae usually includes information on cell diameter, and particularly chloroplast features including shape, length of protrusions, globularity and compressed appearance (Kadlubowska, 1984; Stancheva *et al*., 2012, 2013).

To study chloroplast shape, we applied confocal laser scanning microscopy because this method generates 3D chloroplast images based on autofluorescence without interference from other cellular structures. This method was previously applied in other green algae, e.g. in *Trebouxia* and *Jenufa*, and differences in chloroplast ontogeny (changes during cell growth and autosporogenesis) were described (Škaloud & Radochová, 2004; Škaloud & Peksa, 2008; Němcová *et al*., 2011). We compared the plastid shape of young vegetative cells in fresh exponentially growing cultures (3 weeks after inoculation) for all genotypes, and this was stable in cells grown under controlled laboratory conditions. Vegetative filaments of *Zygnemopsis* are usually indistinguishable from those of *Zygnema* using light microscopy, and the two genera can be separated only by features observable during sexual reproduction (Transeau, 1951). However, based on 3D chloroplast morphology, these two genera were clearly discernible: chloroplasts of *Zygnemopsis lamellata* are porous and include wide, thick lobes with deep incisions, some of which are appressed to the cell wall, whereas *Zygnema* chloroplasts are not deeply dissected and where wide lobes are formed they are flat and do not contact the cell wall. In addition, chloroplasts of *Zygnemopsis* have a more granular appearance. However, it should be noted, that the chloroplasts in different species of *Zygnemopsis* are variable in shape, which may complicate the separation of *Zygnema* and *Zygnemopsis* based on vegetative morphology (Transeau, 1951).

In the investigated *Zygnema* genotypes, differences in chloroplast shapes were apparent. We did not record any phylogenetic signal in chloroplast shape, and assume that chloroplasts of similar shape could have arisen repeatedly during evolution. Chloroplast shape in *Zygnema* is influenced by various factors. For example, during maturation and pre-akinete formation, chloroplasts lose their complex shape with lobes and become smaller (Fuller, 2013; Pichrtová *et al*., 2014*b*; Herburger *et al*., 2015). Thus, all our strains were investigated during exponential growth to avoid chloroplast shape changes caused by life cycle differences or suboptimal conditions. Although chloroplast shape itself is not sufficient for species determination, it may serve as an additional morphological feature upon which to characterize and define species identity. It provides better discriminatory power at genus level to distinguish vegetative filaments of *Zygnema* and *Zygogonium* (Stancheva *et al*., 2014) for example.

In addition, we observed significant differences in cell width among individual genotypes, but they did not reflect phylogeny as previously suggested (Herburger *et al*., 2015). Moreover, the extent of variation in cell width was very high in some genotypes such as *Zygnema cylindricum* (genotype J), which indicates that cell width may not be a reliable feature for discrimination. Cellular diameter is influenced by additional factors such as culture age (Herburger *et al*., 2015) and environmental conditions (Miller & Hoshaw, 1974; Stancheva *et al*., 2012). Doubt was first cast on filament width as a taxonomic character of *Zygnema* by Miller & Hoshaw (1974), who suggested that this feature could be influenced by polyploidy. Polyploidy within the genus *Zygnema* has been proposed several times (Miller & Hoshaw, 1974; McCourt *et al*., 1986), and our cell width data may also support this possibility, but it has not yet been proven experimentally. Polyploidy therefore remains an open question with important implications for taxonomy, because the existence of species complexes complicates species delimitation. For example, the number of *Spirogyra* species is widely regarded as exaggerated, because different ploidal levels within complexes can interbreed (Wang *et al*., 1986).

### Conclusions

In this study, we identified 12 different *Zygnema* genotypes from sampling sites in Svalbard, representing unexpectedly high genetic diversity within putative *Zygnema* mats. Surprisingly, a degree of diversity was also revealed within individual mats that were believed to be formed by a single species. Although the mats appeared uniform, slight gradients in environmental conditions could create microhabitats preferred by distinct genotypes with different biochemical and physiological adaptations. High genetic differentiation may also contribute to the ecological success of *Zygnema* in environments where unpredictable changes might occur. The investigated genotypes were evenly distributed among genotypes of non-polar origin, and did not form any monophyletic polar clusters.

Although most specimens were sterile, we observed conjugation and zygospore formation, representing the first record of sexual reproduction in *Zygnema* and *Zygnemopsis* from Svalbard. Although conjugation is believed to be extremely rare in Polar regions, it seems that at least some species benefit from investment in zygospore production. Although molecular methods are clearly essential for studying diversity in sterile vegetative filaments, our 3D reconstruction of chloroplast morphology using confocal laser scanning microscopy provided sufficient information to distinguish *Zygnema* and *Zygnemopsis* genera. However, chloroplast shape did not appear to reflect the phylogenetic position of *Zygnema* strains. We therefore assume that chloroplasts of a similar shape could have arisen repeatedly during the course of evolution.

In the extreme habitats of Svalbard, *Zygnema* mats contribute substantially to primary production. Knowledge of their species composition, life cycles and survival strategies (including spore formation) may help us to understand their importance at the ecosystem level. Moreover, any structural and functional knowledge could help to resolve the diversity and evolution of Zygnematophyceae, the most species-rich charophyte lineage. Furthermore, this knowledge will improve the use of these algae as model organisms in experimental studies, and facilitate comparisons while reducing the risk of misinterpretation.

## Supplementary Material

Supplementary material 4. Complete results of statistical analyses.

Supplementary material 3. Environmental parameters measured during sampling in 2015.

Supplementary material 2. List of all sequences used in the alignment.

Supplementary material 1. Alignment of the 63 unique rbcL sequences used in phylogenetic analyses.

## Supplementary information

The following supplementary material is accessible via the Supplementary Content tab on the article’s online page at https://doi.org/10.1080/09670262.2018.1476920

**Supplementary material 1.** Alignment of the 63 unique *rbc*L sequences used in phylogenetic analyses.

**Supplementary material 2.** List of all sequences used in the alignment.

**Supplementary material 3.** Environmental parameters measured during sampling in 2015.

**Supplementary material 4.** Complete results of statistical analyses
